# Levels and Norm-Development: A Phenomenological Approach to Enactive-Ecological Norms of Action and Perception

**DOI:** 10.3389/fpsyg.2020.01666

**Published:** 2020-08-11

**Authors:** Miguel A. Sepúlveda-Pedro

**Affiliations:** Department of Philosophy, University of Montreal, Montréal, QC, Canada

**Keywords:** enactive approach, skilled intentionality framework, phenomenology, normativity, affordances, Merleau-Ponty, embodiment, perception

## Abstract

The *enactive approach* and *the skilled intentionality framework* are two closely related forms of radical embodied cognition that nonetheless exhibit important differences. In this paper, I focus on a conceptual disparity regarding the normative character of action and perception. Whereas the skilled intentionality framework describes the norms of action and perception as the capacity of embodied agents to become *attuned* (i.e., skilled intentionality) to preestablished *normative frameworks* (i.e., situated normativity), the enactive approach describes the same phenomenon as the *enactment of norms* (i.e., as sense-making) at different levels of organization that go from individual biological agents to linguistic encounters. I will argue that although both accounts accurately recognize important features of the norms of action and perception, they also have significant shortcomings. Norm-attunement accurately sees normative, ecological frameworks as the necessary set of constraints for the existence of norms at play in sociocultural bodily practices, but it fails to acknowledge the temporal and open-ended character of these norms and frameworks. Norm-enactment, by contrast, acknowledges that norms of action and perception are temporally open-ended, but fails to explicitly recognize that environmental normative frameworks are necessary for the enactment and development of all sort of norms in the interactional domain of an agent-environment system. To overcome these problems, I propose an enactive-ecological approach to norms of action and perception. This approach consists in describing norm-enactment as a result of a developmental process I call norm-development. This process describes the enactment of norms from the background of ecological, normative frameworks. These frameworks are norms enacted in the past of the interactional history of the agent-environment system that remain open to new configurations (new norms) in the present. To clarify conceptually norm-development, I appeal to Merleau-Ponty’s descriptions of *norms of perception*, and more particularly to his concept of *spatial levels*. Like the enactive approach, Merleau-Ponty recognizes that perceptual norms emerge in the interactional history of the agent-environment system, but, like the skilled intentionality framework, he also posits that normative frameworks, that he calls levels, enable and constrain the emergence of perceptual norms and its development. Levels are therefore a phenomenological description of ecological normative frameworks that has been temporally constituted and that stay temporally open-ended as a fundamental requisite for the enactment and development of norms of action and perception.

## Introduction

The enactive approach and the ecological approach of the skilled intentionality framework are two radical forms of embodied cognition that reject the orthodox conception of cognition as a computational function that is physically implemented in brain processes (e.g., Anderson, 2007; Metzinger, 2009). Instead, both the enactive approach and the skilled intentionality framework conceive cognition as an activity rooted in the dynamic sensorimotor coupling of the body and the environment (Rietveld and Kiverstein, 2014; Varela et al., 2016). This coupling permits cognitive agents to establish successful cycles of action and perception in bodily practices (Di Paolo et al., 2017; Rietveld et al., 2018), and to lay the foundation for other, more complex forms of cognition (Di Paolo et al., 2018; Kiverstein and Rietveld, 2018). Despite these shared convictions, there are important differences between these two approaches that prevent their *prima facie* potential complementarity (Chemero, 2009; Heras-Escribano, 2016).

One of the most important discrepancies between the enactive approach and ecological approaches to cognition is Gibson’s claim affirming that ecological information (environmental structures of sensorimotor correlations) exists as a necessary condition for perception (Gibson, 1979/2015). For the enactive approach, perception depends on the enactment of a normative domain of sensorimotor interactions between an agent and the environment (Thompson, 2007). This normative domain is enacted in the concrete history of interactions between each individual agent and the environment, and no pregiven norms are given before this concrete sensorimotor history. The existence of ecological information as a necessary condition for perception is thus rejected by the enactive approach (Varela et al., 2016).

In this paper, I focus on a more contemporary difference that nonetheless recalls the earlier one. This new divergence arises when the supporters of both approaches claim that cognition is a phenomenon based on norms. Whereas the skilled intentionality framework describes skillful action as a process of *norm-attunement* (skilled intentionality), the enactive approach describes the same phenomenon as *norm-enactment* (sense-making). Norm-attunement implies the existence of normative frameworks (situated normativity) toward which individual subjects become attuned to once they acquire mastery of a bodily skill. Norm-enactment, by contrast, describes the enactment of norms based on the concrete history of interactions of the agent-environment system, but without explicitly acknowledging that normative, ecological sets of constraints are necessary for this process.

I will argue that both accounts of norms possess accurate descriptions and explanations of norms of action and perception, but that they also have important shortcomings. Norm-attunement accurately describe the existence of normative, ecological frameworks as the necessary set of constraints for the existence of norms of sociocultural bodily practices, but this description fails to acknowledge the temporally open-ended nature of these norms, and their frameworks. The skilled intentionality framework recognizes that norms change over time due to transformations in the environment and as a result of the purposive activity of agents. However, this approach misses a crucial aspect of all embodied practices: the need for a spontaneous transformation of normative frameworks due to the internal dynamics of the interactional space between and agent (or multiple agents) and the environment. Norm-enactment, by contrast, acknowledges that norms of action and perception are temporally open-ended and, consequently, are open to constant changes in light of the complex dynamics of bodily practices, but the enactive approach fails to explicitly recognize that ecological and normative frameworks are necessary for the enactment and development of all sort of norms in the interactional domain of an agent-environment system.

I propose therefore an “enactive-ecological approach” to norms of action and perception as a way of overcoming these descriptive shortcomings of the skilled intentionality framework and the enactive approach. My proposal is to refine the account of norm-enactment with what I will call *norm-development*. This descriptive model not only conceives of norm-enactment as a temporally open-ended process, but accords with the ecological, normative frameworks that such a process requires.

To clarify this idea of norm-development, I propose to go back to the phenomenological work of Merleau-Ponty. He recognizes that perceptual norms emerge in the interactional history of the agent-environment system, but he also posits that normative frameworks, that he calls levels, enable and constrain the emergence of perceptual norms and its development. From a phenomenological perspective, the concept of spatial levels designates the ecological frameworks that has been temporally constituted and that stay temporally open-ended, fulfilling thus the description of norm-enactment as norm-development.

## Ecological Norms: Normative Frameworks and Norm-Attunement

In the context of cognitive science, normativity usually refers to the correctness or incorrectness of actions based on activities such as perceiving, remembering, imaging, reasoning and so on^1^. The subject of norms and normativity has been one of the main axes of the ecological approach of the skilled intentionality framework because this approach has been always concerned about the way that individual cognitive agents acquire the required skills to participate in sociocultural practices (Rietveld et al., 2018). In this regard, the skilled intentionality framework has two different but interrelated descriptions of norms and normativity: (1) *situated normativity* (Rietveld, 2008), and (2) *skilled intentionality* (Bruineberg and Rietveld, 2014). In what follows, I will unpack these two fundamental concepts.

### Situated Normativity

The notion of situated normativity is motivated by Wittgenstein’s accounts of how a skillful agent is moved to take a particular set of actions to produce a satisfactory outcome of a sociocultural practice. Skillful agents, like tailors and architects, for instance, feel discomfort and discontent if they find the conditions of their practices unsatisfactory (Wittgenstein, 2007). If they have enough expertise, they can be moved to take action to improve these conditions (Rietveld et al., 2018). This can happen without the need for conscious reflection, because it is the feeling of dissatisfaction, and the felt demand or solicitation to take a particular set of actions, that actually describes the lived experience of skillful agents in action (Rietveld, 2008). This description reveals that skillful agents are already attuned to a normative framework that is not individual or private, but social and public. Sociocultural practices like tailoring and architecture have standards that are explicitly or tacitly accepted by a community to which tailors and architects belong. Thus, feelings of dissatisfaction and solicitations of action are grounded on public standards. This description led scholars in the skilled intentionality framework to adopt the idea that situated normativity does not refer to norms enacted by individuals, but to norms that rule the habitual patterns of bodily practices of a sociocultural group. These patterns were called, after Wittgenstein, a *form of life* (Rietveld and Kiverstein, 2014; Rietveld et al., 2018).

A form of life does not occur in a vacuum though. They are entangled with material structures or physical constraints that help to constitute and shape the norms of practices. For this reason, the skilled intentionality framework designates the environmental conditions of a human practice as a *sociomaterial environment* (van Dijk and Rietveld, 2017). Hence, there is a sociomaterial entanglement in the form of life of human beings. The paradigmatic case of a form of life is a sociocultural human group, but the notion of a form of life, nonetheless, is not exclusive to human beings. Human and non-human animals’ forms of life inhabit spatial regions that can be described as *ecological niches* (Rietveld and Kiverstein, 2014). These niches are not simply the raw material composition of a spatial region, rather these niches are best seen as the entanglement of this materiality with a form of life; indeed, an ecological niche refers to the whole set of a *landscape of affordances* for a form of life (Bruineberg, 2018).

The notion of affordances was originally defined by Gibson as the possibilities for action that the environment affords to an animal, for good or for ill (Gibson, 1979/2015). Although affordances are perceived in the environment, they cannot be understood without reference to the animal that perceives them, and for this reason it has been argued that affordances are relational properties of the animal-environment system as a whole (Gibson, 1979/2015; Warren, 1984; Heft, 1989). Chemero has argued, nonetheless, that affordances are more than relational properties: they are relations between the bodily skills of an animal and the relevant “features of the environment” (Chemero, 2009). This is because affordances are only perceived by animals that possess the required bodily skills to exploit the resources the environment affords, and because affordances do not refer to properties of objects but to the contextual conditions of a situation.

For the skilled intentionality framework, however, affordances should not be understood as relations between an individual animal and the environment, but between a form of life and the material (and in the case of humans, the sociomaterial) environment (Rietveld et al., 2018). The skilled intentionality framework distinguishes between two different sets of affordances: the first being a *landscape*, and the second being a *field of affordances* (Rietveld and Kiverstein, 2014). Whereas a landscape of affordances represents all those affordances available to a form of life, a field of affordances refers to a subset of this landscape, composed of affordances relevant for the task of a skillful agent. Such affordances can be seen as the *solicitations* that move an agent to act (Rietveld et al., 2018). As a result, an ecological niche entails the whole landscape of affordances of a form of life. In the case of non-human animals, the ecological niche is the relation between the patterns of behavior of a species, and the material conditions of their environment. In the case of humans, the ecological niche is the relation of patterns of behavior of a sociocultural group and the broader sociomaterial environment. In both cases, the normative framework is defined in reference to a group of individuals, and not to individuals as such.

### Skilled Intentionality

The notion of skilled intentionality is built on two main pillars: Merleau-Ponty’s phenomenology and Friston’s free energy principle (Bruineberg and Rietveld, 2014). Skilled intentionality relates the process of attunement of a skillful agent to the relevant affordances. Phenomenologically, skilled intentionality can be described as the movement of a body toward an optimal equilibrium of the practical situation, or toward what has been called an optimal grip (Bruineberg and Rietveld, 2014). This tendency was originally defined by Merleau-Ponty in the following paragraph:

For each object, just as for each painting in an art gallery, there is an optimal distance from which it asks to be seen – an orientation through which it presents more of itself… The distance between me and the object is not a size that increases or decreases, but rather a tension that oscillates around a norm (Merleau-Ponty, 2012, p. 315–316).

Following Merleau-Ponty, the skilled intentionality framework sees skillful agents as sensitive to the adequate affordances in a situation, and this sensitivity entails the capacity of agents to take the required action to change the equilibrium of a situation, bringing it closer to its optimal state. This supposes that each practical situation entails a norm or an optimal state that is dependent on the goals of individuals, as well as on conditions in the sociomaterial environment (Bruineberg and Rietveld, 2014).

The distinction between a field of affordances and a landscape of affordances is crucial, because whereas the landscape of affordances is defined by situated normativity, the field is better defined by skilled intentionality. The field of affordances, contrary to the landscape, is dynamic and can change at multiple temporal scales. At the behavioral scale, for instance, during the execution of a practice, the actions required to reach the optimal grip change constantly, because of the dynamic change of the practice itself (Bruineberg and Rietveld, 2014). At the developmental scale, the change of interests of an individual and changes in the material conditions of the environment can alter the relevant affordances (Chemero, 2009). At the sociohistorical scale, the nature of the practices, for example the customs and traditions, can also change, altering the field of affordances (Malafouris and Renfrew, 2013). Therefore, we can view skilled intentionality as a more flexible and dynamic description of normativity than that found in situated normativity.

Nevertheless, for the skilled intentionality framework, skilled intentionality and situated normativity are interrelated and complementary. Situated normativity, and the concept of the landscape of affordances, describes subject-independent aspects of norms of cognition. Skilled intentionality and its field of affordances describes the more contingent and subjective aspects of these norms (Rietveld et al., 2018). However, the dynamic development of a field of affordances can also alter the conditions of the environment, producing a dialectical movement between the agent and the environment that constantly alters the field of affordances (Bruineberg et al., 2018). Following theories of self-organization, the skilled intentionality framework shows how the conditions of the environment can constrain the self-organization of a system and reveals how the processes of self-organization can alter environmental conditions. This produces an effect of circular causality, where agents and environments become entangled because they are mutually constrained (Bruineberg and Rietveld, 2014). The result of this dynamic movement is the constant change of the field of affordances.

The naturalization of skilled intentionality by proponents of the skilled intentionality framework succeeds thanks to Friston’s account of the free energy principle (Friston, 2010). This principle offers a statistical and dynamical model for understanding how the brain-body-environment system organizes itself to reduce uncertainty, or what is known as *variational free energy^2^*.

Uncertainty, or variational free energy causes an organizational disequilibrium in the brain-body-environment system that is affectively felt by cognitive subjects as a bodily tension that must be reduced (Bruineberg and Rietveld, 2014). The organizational composition of the body and the brain allows subjects to modulate their coupling with the environment in order to reduce variational free energy by a process called *active inference*. Active inference can produce changes in the system that reorganizes the brain, body, environment system, thanks to processes of motor action (Bruineberg et al., 2016).

The tendency to reduce affective tension explains the movement of the body to reach the optimal grip. This activity directed toward the optimum can either occur by changes in the self-organization of the brain-body system or by changes in the structure of the environment. The optimum is thus a norm that tacitly leads agents’ behavior and their perception of affordances.

In sum, the skilled intentionality framework holds two different accounts of norms and normativity. On the one hand, situated normativity describes normative frameworks of social and biological groups, while on the other hand, skilled intentionality describes the more concrete attunement of individuals to those normative frameworks. I will call the first phenomenon *normative frameworks* and the second *norm-attunement*.

## The Norms of Life and Cognition

The enactive approach rejects the traditional definition of cognition as information processing and proposes instead an understanding of cognition as a form of *sense-making* (Di Paolo and Thompson, 2014). Sense-making basically implicates the enactment of normative domains of interaction between an agent and the environment. There are four main forms of sense-making related to four different levels of agency: the vital, the sensorimotor, the intercorporeal, and the linguistic levels, In what follows, I shall unpack the main aspects of the different forms of sense-making and the norms of cognition, according to the tenets of the enactive approach.

### Vital Norms

For the enactive approach, life and cognition share the same type of formal organization (Thompson, 2007). Cognition is a more complex form of the basic modes of interaction of living organisms and their environments, and it is for this reason that any account of cognition must be derived from the basic descriptions of life (Di Paolo et al., 2018).

In this regard, the enactive approach sees living organisms as autonomous systems in precarious conditions with adaptive behavior (Di Paolo and Thompson, 2014). They are autonomous systems because organisms are systemic wholes of interrelated processes that have *organizational closure* (Varela, 1979). This means that living systems are composed of a network of processes that are causally interdependent, allowing living systems to constantly produce and maintain networks of processes (Di Paolo and Thompson, 2014). As with any other physical system, living systems increase entropy with time (Ruiz-Mirazo and Moreno, 2004), risking the loss of their autonomous organization, and ultimately, death (Weber and Varela, 2002; Di Paolo, 2005). To avoid destruction, organisms need to exchange matter and energy with their surroundings through processes of metabolism. As such, organisms should be understood as thermodynamically open systems that constantly renovate their material components to assure the viability of the system (Di Paolo, 2005). To accomplish these interactional processes with the environment, organisms must adapt or modulate their behavior according to norms that allow the system to remain viable (Barandiaran and Moreno, 2008). This means that the environment is primarily disclosed to organisms in light of their own fundamental concerns, which can be understood as moving away from destruction (Di Paolo and Thompson, 2014). Vital norms are thus norms that allow living organisms to satisfy biological needs and maintain *viable* their autonomous organization.

It should be noted that even this basic form of sense-making is more affective than purely cognitive, because the way the environment is disclosed by an organism is related to how the environment causes affective bodily states in the organism (Colombetti, 2014). This is to say that it is the body-environment state of organizational disequilibrium, and not the environment as a neutral landscape, that is felt by the living organism. This basic affectivity of life is akin to the affective state of humans described by Damasio (1999) as the feeling of being alive, which implies all of the brain activity related to the basic regulatory processes of the body. This feeling of being alive is arguably the basic requirement for any kind of sense-making and cognition (Fuchs, 2018). Therefore, for the enactive approach, the norms of cognition involve fundamentally affective states, and not merely cognitive states.

### Sensorimotor Norms

At the sensorimotor level, where properly speaking, cognition appears (Barandiaran, 2017), a new form of sense-making arises thanks to the self-organization of the brain-body-environment system. This process of self-organization allows living agents to interact with the environment to accomplish practical tasks, from fulfilling biological needs, to tasks unrelated to these basic needs (Di Paolo et al., 2017).

Sensorimotor interactions are based on patterns of self-movement correlated to changes in the sensorial field. These correlations are known as *sensorimotor contingencies* (O’Regan and Noë, 2001). When these sensorimotor contingencies involve the coordination of many parts of the body, including brain activity, they are called *sensorimotor coordination* (Buhrmann et al., 2013). When the coordination of the brain-body-environment system accomplishes a determinate practical task, implicating a normative outcome, sensorimotor contingencies implicate processes of self-organization called *sensorimotor schemes* (Di Paolo et al., 2017). These schemes are formed and reinforced by the successful realization of tasks, forming clusters of interdependent schemes that create the bodily or sensorimotor *habits* we observe in our everyday tasks. These tasks are given in specific contexts that solicit the enactment of a whole set of interrelated habits, establishing what the supporters of the enactive approach call a *microworld* (Varela, 1999; Di Paolo et al., 2017). Thanks to this self-organization of habits, the living body of cognitive agents acquires a new identity, a “sensorimotor self” that becomes different from the self-identity of life, because this new self is constituted by particular *sensorimotor norms* (Di Paolo et al., 2017, p. 142).

Although these sensorimotor norms can be rooted in biological needs, such as when human and non-human animals look for food and shelter, they can be also founded on the incorporation of sociocultural practices, such as cooking a dinner or dancing. Nonetheless, even if sensorimotor norms are originated in social frameworks rather than in the biological activity of the body, such norms need to be incorporated by the living body to enact meaning or relevance for the body’s interactions with the environment (Di Paolo et al., 2017).

### Intersubjective Norms

The third relevant form of sense-making and normativity articulated by the enactive approach is the enaction of norms that occasionally emerge from the interaction of two or more autonomous systems, called *participatory sense-making* (De Jaegher and Di Paolo, 2007; Froese and Di Paolo, 2009). When two or more autonomous systems interact, they often need to coordinate bodily movements in a way that allows each system to adapt its bodily self-organization for the accomplishment of a common goal. On some occasions, these interactions can produce a pattern of coordination that constitutes an emergent form of self-organization that becomes partially autonomous in relation to the purposes of individual participants.

The effect of this emergent self-organization of the interactive system forces individual participants to modulate their own sensorimotor norms, producing conflict between two different levels of normativity: the individual, and the collective (De Jaegher and Di Paolo, 2007). Such readjustment of the norms of individuals, caused by the emergent participatory system, allows these individuals to acquire new forms of sense-making, that is, new normative ways of interacting with the environment. These emergent participatory norms cannot be achieved individually, because it is only in the interaction with another participant that such forms of sense-making can be enacted (McGann and De Jaegher, 2009). However, they can permanently alter the sensorimotor norms of individuals even if they are not actively engaged in a participatory practice (Di Paolo et al., 2018). Therefore, for the enactive approach, there are norms of sensorimotor interactions that exceed the autonomy of individual living beings because these norms are enacted in a system of coordination that is composed of more than one individual.

### Linguistic Agency and Social Normativity

The model of participatory sense-making has now moved one step forward and describes the emergence of a new form of agency that can fully account for the normativity at play in sociocultural bodily practices (Di Paolo et al., 2018). This is a linguistic agency that emerges thanks to the permanent tension at play in the social interactions between individuals and social norms. This primordial tension can produce metastable processes that can function as instruments for the coregulation and meta-coregulation of social coordination, and eventually, to the use of public utterances that open a linguistic dimension for participants in a community. Although this model is too complex to be fully outlined here, it is enough to bring forth its main features to illustrate how, for the enactive approach, different degrees of social normativity emerge in the dialectics of participatory sense-making.

The original model of participatory sense-making already exhibits a permanent tension between the individual and the social or interactive levels of normativity. In the updated model of participatory sense making, this tension remains constant through different stages of conflict (dissonance) and harmonization (synergy) between the two levels of normativity. This tension initially forces individuals to adjust their own sensorimotor norms (sensorimotor regulation), but eventually such adjustments must be carried out jointly (sensorimotor coregulation). The sensorimotor coregulation of social interactions eventually produces social acts that serve to make these coregulatory acts more efficient. This is a process of meta-coregulation that will be present all across the following stages of the enactive model.

The efficiency of social acts of coregulation and meta-coregulation in wider social groups lead agents to the mutual recognition of other participants as agents. This becomes explicit in the emergence of a dialogic interaction, where the roles of an active regulator and a passive regulated member are interchangeable. At this dialogical level, agents use utterances to regulate social interactions, and there is a progressive construction of dialogical networks of utterances that are shared by a community in particular contexts of bodily actions, one that Di Paolo et al. (2018) call *participation genres*.

Participation genres bears similarities to the notion of micro-worlds at the sensorimotor level of autonomy, although in the former case, the normative structures of interaction involve not only networks of dynamic sensorimotor processes, but also a network of utterances.

Although these networks are constantly regulated by processes of mutual interpretation between multiple participants, these regulations may also take the form of self-interpretation. This can occur when a user of utterances becomes aware of an impairment between the pragmatic and expressive aspects of her own utterances (e.g., the utterance does not produce in others the responses she is expecting, according to what she is trying to express). This moment is crucial in the enactive model, because a new level of reflective, dialogical dynamics is incorporated to the intersubjective skills of an agent. The successful utterances become regular patterns of dialogical practices for individual participants (either for their interactions with other agents or for their own interactions with the environment). The norms, co-enacted with others and embodied in networks of utterances (participation genres), now play a more explicit role as tools for self-regulation. Utterances are incorporated by individuals as regulatory tools for their expressive and pragmatic goals, and the new dialogical networks, afford the possibility of making explicit and questioning the already existent normativities at play. This gives to agents the opportunity to dialogically reshape and move forward the already existent norms.

The enactive approach has been criticized for being incapable of explaining social normativity because its account of vital, sensorimotor, and intercorporeal forms of sense-making refers exclusively to the normative domain of individuals (e.g., Heras-Escribano et al., 2015). Now, however, this approach offers a theoretical sketch of the emergence of social norms as arising from tensions inherent to the social interactions of autonomous agents. Recognizing situated normativity in this long and complex model of the enactive approach is not easy and requires an analysis that exceeds the scope of this paper. However, it is possible to recognize in this model how individual agents progressively acquire new regulatory processes that emerge from social interactions. It is at the final stages that the actions of agents are more explicitly guided by norms that are social and public, but from the early stages, individual agents are constrained by norms that are jointly enacted by more than one individual.

We can therefore conclude that the most relevant notion of norms found in the enactive approach can be located in the emergence of an interactional domain between one or many agents and its environment. These sense-making norms continue unfolding in time, according to constraints located in the history of interactions of the agent-environment system (Thompson, 2007; Varela et al., 2016). This descriptive account of norms is what I shall call *norm-enactment*.

## Enacting Norms or Following Rules?

In traditional cognitive science, perception consists of simply retrieving information from the environment, thanks to the brain’s capacity to produce internal representations or models from sensorial stimuli. It is assumed in these models that the facts of the world are independent of the subject, and the role of cognitive systems is simply that of accessing this ready-made reality. From this perspective, if cognition and meaning are normative, it is only because the contents of internal representations are more or less accurately correlated to the facts of a ready-made world (e.g., Millikan, 1984).

Neither the enactive approach, nor the skilled intentionality framework assumes that cognition consists in the production of internal representations of a ready-made world (Di Paolo et al., 2017; Rietveld et al., 2018). Rather, for these approaches, it is the active engagement of embodied agents that makes possible the experience of a meaningful world. Sensorimotor interactions establish the primordial link between agents and the environment, and it is in this domain that fundamental norms of interaction and perceptual meaning emerge. The primordial layer of perceptual meanings is better understood as the opportunities for action that the environment provides for the accomplishment of sensorimotor tasks. However, these perceptual meanings depend on the bodily skills of agents. Although this basic picture works for the enactive approach and for the skilled intentionality framework, there is nevertheless one fundamental divergence in their claims that should call our attention. Whereas the enactive approach describes the constitution of cycles of action and perception in individuals as *the enactment of norms*, the skilled intentionality framework describes them as *the attunement of individuals to pregiven normative frameworks*. In my view, both accounts of norms have relevant shortcomings on this account.

Norm-attunement sees normative frameworks as pregiven sets of constraints that shape the embodiment of skillful agents, but these constraints are not themselves reshaped by dynamic processes of embodiment. The normative frameworks described by situated normativity are not eternal nor unmovable; they change as a result of transformations in the material structure of the environment and in the body (Bruineberg and Rietveld, 2014). Ultimately, they can change as a result of the intervention of agents possessing higher forms of cognition (Rietveld et al., 2017, 2018). Significantly, these descriptions leave out, however, one crucial aspect of all bodily practices (social or not), and it is this: the incessant transformation of norms due to the dynamical interaction of agents and the environment.

Norm-enactment, by contrast, fails to explicitly acknowledge the central role that ecological (normative) structures play in constraining the interactional domain of an agent(s)-environment system. These ecological structures, we must be clear, are not raw physical structures, but material structures impregnated with meaning. These environmental structures are not only necessary for explaining the enactment of norms, but also for understanding their progressive development.

Ecological and normative frameworks, however, are temporally open-ended structures that may constantly change due to tensions and ever-present movement within bodily practices. I will describe later how these sorts of temporally open-ended and ecological structures are necessary for the enactment of norms. I will call the descriptive account of the enactment of norms that includes these type of structures *norm-development*.

### Breaking the Rules: Creativity and Improvisation

As we’ve seen, the norm-attunement element of the skilled intentionality framework offers a well-grounded theory of how, from a third-person perspective, individual agents incorporate the normative standards of a community for the realization of a bodily practice. This approach accurately recognizes that to explain the normative regulation of bodily sociocultural practices, normative frameworks, based on social conventions, are required. Norm-attunement, however, does not acknowledge that the constitution and progressive development of social norms are not extrinsic to the active participation of individuals. The process of incorporation of sensorimotor norms (social or not) actually implies an internal dynamic movement that causes a self-movement, or a natural development of norms. Let me illustrate this phenomenon with the paradigmatic case of jazz improvisation.

Jazz improvisation requires some normative frameworks that jazz musicians respect, such as harmonic shifts and progressions (Walton et al., 2015). Jazz standards also provide a framework for improvisation. The personal style of each musician embodies their own normative way of playing jazz (Sawyer, 1992). Nonetheless, the improvisation – a good one at least – entails a dimension where all these norms are structures allowing agents to engender new ways of expression (Montuori, 2003), that is, new norms. This is true first of all because jazz improvisation consists in renewing the normative framework of jazz standards according to the current conditions of the environment. Such an environment may include the emotional states of participants, their interactions (Linson and Clarke, 2017), as well as the public (Sawyer, 1992; Walton et al., 2015). However, it is important to see that the success of an improvisation (or acting according to a norm) consists in doing the same thing, but always in a new way (Schiavio and Cummins, 2015), i.e., in a way that breaks pre-established rules (Barron, 1963). Successfully establishing a new norm in an improvisation (a new way to do things right) is more often than not a pre-planned action. Rather, success is the result of enacting a new “sense” when agents are immersed in the dynamics of the practice (Walton et al., 2015). This does not mean that jazz improvisation consists in unreflective action, since reflective and unreflective actions can be at play (Sawyer, 1992) at the same time. Instead, committing errors or breaking rules in unpredictable ways allows agents to reshape the pre-established normative framework and thereby enact new norms (Montuori, 2003; Walton et al., 2015).

In jam sessions, improvisation already exhibits an open-endedness in its interactional norms, but the dynamic “self-movement” of this practice goes further. In these sessions, musicians constantly and collectively create new structures of sense (new melodic patterns, licks, riffs, etc.) from previously given structures (standards, personal styles, musical rules). Some of these new melodic patterns are successful and may become part of the habitual repertoire of one or more of the practitioners. These new musical patterns are also open-ended structures, because even as repetitive musical phrases, they nonetheless vary all the time. The accumulation of new musical patterns can eventually transform and update the current personal norms of each musician (a personal style), as well as the norms of the particular collectivity (a group’s style). The personal style of a musician, or the collective style of an ensemble can acquire a level of success that influences musicians in a wider community, and create a whole new style (e.g., Miles Davis and the birth of Cool Jazz). Once again, all along this process, reflective and unreflective actions take place, but the transformation of an old social norm (let’s say Bebop) into a new one (e.g., cool jazz) cannot happen if musicians do not break (intentionally and unintentionally) previously given norms.

Jazz improvisation is a paradigmatic example of interactional dynamics moving forward the development of norms because, for jazz practitioners, spontaneous creativity and novelty is an explicit command for the right accomplishment of this practice. This creativity nonetheless is not an aesthetic luxury for other bodily practices, it is a necessity.

The use of recurrent and historically acquired patterns needing constant adjustments to deal with new circumstances takes place in all sorts of bodily practices. Baber et al. (2019) for instance, describe how goldsmiths need to constantly adjust and adapt previously acquired techniques, in light of the type of “responses” the material sends back to their bodily actions. This dynamic process of re-adaptation involves a continual re-interpretation of the space of available affordances, one that keeps changing insofar as the process of making jewelry continues. The expertise of a goldsmith actually consists in being capable to make the proper adjustments of their habitual patterns of interaction to suit contextual demands.

Likewise, Ingold (2010) claims that many social practices consist in the constant adaptation of action to the constant flow of both material and forces. For Ingold, the paradigmatic example of bodily practices is textile weaving, whereby weavers use available material to design a unique path of becoming, one that embodies the context of the weavers. Ingold contrast how weavers deal with the dynamical flow of materials with modern architecture. Ingold claims that modern architects are distant from the process of building, and instead of dealing with the flow of materials, they reflectively imagine and plan the form and shape that materials will acquire. This modern practice is in sharp contrast with that followed by medieval architects. The architect responsible for the cathedral of Chartres, for instance, was the master of builders, and, as such, he stayed on site in the building process to deal with the contingencies of his endeavor. In this case, there was actually no plan in advance, and the final outcome was the result of the process of dealing with both the available material, as well as contextual demands (Ingold, 2010).

The status of architecture has been a major concern for supporters of the skilled intentionality framework. Contrary to Ingold, I do not think contemporary architecting is a disembodied practice as he describes it. The skilled intentionality framework has convincingly argued that architecture is tightly connected to affective bodily sensitivities (Rietveld and Brouwers, 2017; Rietveld et al., 2017). This argument is helpful in closing the gap between basic and complex forms of cognition (Rietveld et al., 2018). For the practice of architecture, the complex entanglement of different bodily actions involving the use of many cognitive and technological resources needs further analysis, so that we may understand how a contemporary architect deals direct or indirectly with the material flow. Be this as it may, I am convinced we should not describe architecture as a disembodied practice, but rather as a very complex form of embodied and enculturated practice.

It is important to understand that the dynamic development of norms is not restricted to social practices. Social interactions between agents and technological artifacts such as tools make the internal dynamics of bodily practices more complex, but in the direct relation between a solitary agent and the environment, there is already a need for constant adjustment of sensorimotor norms. I will describe these processes in section “Sensorimotor Development.”

In sum, whether social or not, bodily practices involve a dynamic encounter between the habitual past and the unexpected demands of the environment in the present. For this reason, norms of action and perception are always subject to a continuous development. How practitioner adapt to the contingencies of the present does not always entail significant change, so we can assume that a static set of norms are at play in many practices over long periods of time. This does not mean that the permanent dynamics of bodily practices are not at work. It is precisely for this reason that norms of action and perception should be seen as temporally open-ended norms, subject to changes in the endogenous interactions of a bodily practice. This is an aspect that the skilled intentionality framework fails to acknowledge in their descriptions of norm-attunement.

### The Self-Movement of Norms

The enactive approach offers a more accurate description of the temporally open-ended nature of norms than the skilled intentionality framework. Norm-enactment not only acknowledges the constant dynamic adjustment of norms in light of environmental contingencies, it also sees the constitution of social norms as a dynamic process of tensions and coregulations between autonomous agents that involves the constant movement and development of norms. This does not save the enactive approach from an important shortcoming. This is the neglect of the role that ecological structures can play in the constitution and development of norms (cf. McGann, 2014).

Sense-making is the enactment of norms at the biological, sensorimotor, and social scales. At the biological level, in the paradigmatic descriptions of vital norms, it is clear that these norms are constrained by the physical conditions of the environment. At the same time, it is not equally clear if they are constrained by precedent normative frameworks as well. In the classical example of the enactive approach, where *E. coli* bacteria respond behaviorally to the presence of glucose, it is argued that bacteria make sense of this chemical compound according to their metabolic needs (Thompson, 2007, p. 74), thus bacteria make sense of the glucose as food.

This description suggests that, for the enactive approach, meaningless physical matter acquires meaning thanks to the interests (teleology) of the organism. In this regard, De Jesus (2018, p. 873) criticizes the enactive approach for what he names an “epistemic perspectivalism” of this approach. He argues that sense-making involves the description of the world “in itself” that appears differently (as meaningful worlds) to subjects with different bodies. Such a picture described by De Jesus, however, presupposes a distance between the environment and the agent that is surmounted epistemologically. This description of sense-making is inaccurate.

For the enactive approach, the world described by physics and chemistry (e.g., glucose) is not a description of the world-in-itself, or an objective reality independent of any agent as it is for mainstream scientific approaches. Enactivists see the descriptions of science as part of the meaningful world of humans, and as the result of an embodied and enculturated practice. For the supporters of the enactive approach, scientific descriptions are not descriptions of an objective world (Thompson, 2016). Therefore, the meaning “food” for bacteria, and what a scientist conceives of as “glucose” are not two epistemic perspectives of the same object. Instead, they are two different forms of an agent-environment entanglement.

We need to be clear that the birth of norms is not the result of putting in connection two alien objects, but a reorganization, a new sense of a pregiven form of an entanglement already at play. When we describe the emergence of a vital norm, like *E. coli* bacteria perceiving glucose as food, we cannot state that an organism projects meaning on a raw physical substance. It is more proper to say that an organism *incorporates* an aspect of the environment into its interactional domain (*Umwelt*). An incorporation, in this case, means a reorganization of the agent-environment system, the acquisition of a new sense or a new norm for the sort of interactions this system maintains. For instance, Barandiaran and Moreno (2008) posits that normativity, at the level of life, entails two different kind of processes: constructive processes and interactive processes. The first set of processes consists in the network of processes needed to maintain the autonomous organization of a living organism. They are topologically localized into the boundaries of the organizational closure of the system. The second set comprises the processes of interaction between the agent and the environment that are needed to maintain the viability of the system. It is important to note that both set of processes are necessary to preserve the viability of the system; i.e., both constructive and interactional processes are constitutive of the vital norms of a living organism.

As part of one single system, any change in the norm of interactions means a reconfiguration of the whole system (Gestalt). This is precisely what happens when *E. coli* bacteria find lower levels of glucose and high levels of lactose. The bacteria change its constructive processes (its gene expression) to metabolize lactose instead of glucose, adapting their interactional processes to the current conditions of the environment (Barandiaran and Moreno, 2008). In this case, the adoption of a new norm consists in a reconfiguration of the whole Gestalt, and not simply on the way the agent makes sense of the environment. Since the acquisition of a new norm implies changes in the body of the living agent, or in its constructive processes, the adaptive behavior of an agent also entails some sort of incorporation.

It is common to speak about incorporations in the literature of the enactive approach when human agents change their sense-making capacities through the habitual use of tools (Di Paolo, 2009; Thompson and Stapleton, 2009). This is called *tool-incorporation* (Fuchs and De Jaegher, 2009). There is, however, another sort of incorporation that occurs when other living agents transform our sense-making. This is called *mutual incorporation* (Fuchs and De Jaegher, 2009). There is a third form of incorporation that is not the integration of an environmental aspect into the boundaries of the body, but the incorporation of aspects of the environment into the perceptual field of agents and that are not necessarily affordances. This is something I will call *excorporations*, and I will explain their relevance in the section that follows.

### Excorporations and Norm-Development

The term excorporation was coined by Merleau-Ponty scholar David Morris to describe, from a phenomenological stance, those aspects of the environment that are vital for the body, but that remain external to the body (Morris, 2004, p. 131). He describes excorporations as the counterpart of bodily habits topologically situated in the environment. The idea of excorporation is similar to that of affordances in the language of ecological approaches, but different because the term does not refer to specific practical meanings of things, but to anchorage points of the environment that allow agents to become situated in place (to reside or to inhabit it).

Contrary to incorporations, which are portable aspects of the environment (e.g., the cane of a blind person) excorporations are not portable and remain situated in places (e.g., the door frame of my bedroom). As a result, they appear to be subject independent, but they are not. They are the counterpart of bodily habits, or, better yet, bodily habits are the counterpart of the places a body inhabits. As an example, Morris describes how Earth excorporations are constitutive aspects of the way we inhabit as bodily agents of our planet (Morris, 2004). A further analysis of excorporations can be revealed by examining the notion of spatial levels in Merleau-Ponty’s phenomenology. I will come back to this subject in the last section. For the time being, we need to understand that the idea of excorporation shows us that the environment is entangled to the body in such a way that we must stop thinking of the agent and the environment as two separated objects that become linked only when a sense-making norm arises. The agent-environment entanglement always precedes the enactment of a norm. This enactment is a reorganization or a reconfiguration of the agent-environment entanglement, and the actualization of a pregiven norm.

For this reason, the analysis of sense-making should show us that the enactment of a norm is the result of the actualization of the historical past (a pre-given norm) of the agent-environment system in the current flow of the present. In this case, actualization does not mean a mere adaptation or a transformation of the historical past into the present conditions, as when we change our old-fashioned clothes for the latest fashion designs. Actualization means the conflict arisen from the encounter of bodily habits and the unexpected conditions of the present. This encounter produces a disparity or a tension between the past and the present, making the agent-environment entanglement move forward while engendering concrete living acts that constantly reorganizes the agent-environment entanglement (see Morris, 2017 for a phenomenological interpretation of this phenomenon).

Somehow, every action and perception cycle are the enactment of a new norm, or at least, its actualization (you could not step in the same river twice). But the tendency of agents to reduce the tension created by the disparity between the habitual past and the unexpected present produces a stabilization or a balance that normalizes agent-environment interactions. However, when the disparity creates an important amount of tension, a major reconfiguration of the entanglement is needed, and the enactment of a fully-fledge new norm occurs, which can be understood as a new normalization of the interactional agent-environment domain. Piaget’s theory of equilibration, evoked by the enactive approach to explain the development of sensorimotor norm (Di Paolo et al., 2014, 2017), resonates with this conception of norm-development.

### Sensorimotor Development

For the enactive approach to sensorimotor norms, *Piaget’s theory of equilibration* illustrates the adaptation and transformation of sensorimotor schemes (see section “Skilled Intentionality”) for generating new ways for these schemes to function, when agents find new challenges in the environment (Di Paolo et al., 2017). Two processes are crucial here: *assimilation* and *accommodation*.

Assimilation refers to the integration of an environmental aspect into the physiological or cognitive/behavioral structure of the agent (Di Paolo et al., 2017), and, in the words of scholars of the enactive approach, this is “one way of saying that the agent and environmental sides of a sensorimotor scheme are in agreement according to the relevant norm” (Di Paolo et al., 2017, p. 84). This resonates with what I have called an incorporation of the agent-environment entanglement.

Accommodation, on the other hand, describes the processes by means of which an agent modulates its physiological and/or behavioral structures to facilitate the assimilation of an aspect of the environment that is not yet assimilated. Equilibration, thereby, is the process by which a sensorimotor organization reaches a new stability, reducing the tension and the disparity caused by the encounter of the novel. The result is thus a dialectical process that transforms the past into a new present, reducing the tension between the two, and engendering a new norm. This is what I mean by norm-development.

The only aspect that needs to be reconsidered in this theory is the idea that the enactment of a new norm involves a modulation or an adaptation of the body of an agent and its pattern of behavior, without considering that changes in excorporations also occur. These changes transform the sense of a situation and, consequently, change the specific meaningful aspects of the environment. For instance, learning to swim can be understood as the acquisition of a new skill that comprises multiple sensorimotor schemes (e.g., kicking, stroking, and breathing). In this example, the water of the pool excorporates a sort of place where I can find affordances for floating, diving, toppling, etc. This new agent-place entanglement becomes pregnant with a new realm of possibilities for learning different swimming styles, explorations, dancing, etc. Before the basic swimming-norm was acquired, the water of the pool was not a place of residence, nor it was imbued with a rich landscape of affordances and solicitations. Instead, the pool was a place where doing things in-the-water were senseless.

Norm-development is thus the result of enacting norms, and not a process of following static rules. For this reason, the notion of sense-making is more adequate for understanding the norms of perception than notions of situated normativity and skilled intentionality^3^. The descriptions of the skilled intentionality framework and ecological approaches are nonetheless quite useful for explaining the nature of what I’ve been calling excorporations. A field and a landscape of affordances are useful concepts for understanding the counterpart of bodily habits which has been the focus of the enactive approach. Nonetheless, a truly enactive interpretation of these concepts is needed. As a first step to understanding the ecological realm from an enactive perceptive, I will describe the account of norms and spatial levels found in Merleau-Ponty’s phenomenology. This should help us to understand the logic of norm-development from a full-fledged enactive-ecological approach.

## Norm-Development and the Dialectic Movement of Levels

In this last section, I specify the characteristics of norm-development, in light of the normative account of perception described by Merleau-Ponty (Merleau-Ponty, 2012, 2013), with a special focus on the notions of *spatial levels* and *levels shift* (Talero, 2005; Marratto, 2012; Morris, 2017, 2018). In the first section, I will introduce the context of perceptual norms from the standpoint of phenomenology. In the second section, I will refer explicitly to the notion of levels and, in the last one, how levels shift involves a phenomenological description of norm-development.

### Horizons and Virtual Fields

Phenomenology comes with its own conception of norms that must be clarified before we put forward a phenomenological account of norms of perception that can productively dialogue with the enactive approach and the skilled intentionality framework. I will start by sketching out the normative character of experience in the context of phenomenology.

Phenomenology describes and analyzes subjective experience, but phenomenology is not a description of the contents of our subjective experience. Instead, phenomenology aims to describe and analyze the structural aspects, or the invariants of experiences (Gallagher, 1997). In this regard, phenomenology is a *transcendental philosophy* because it is concerned with the conditions of possibility for having experiences, i.e., for those necessary structures that constitute our perception, remembering, thinking, and so on.

To accomplish a transcendental analysis of experience, Husserl applied a strategy known as the phenomenological *epoché* (Husserl, 1982, p. 61). This epoché puts aside any judgment about the positive existence of the objects we experience, something we spontaneously do in our everyday lives and even in our scientific claims. Husserl called this the *natural attitude* of experience (Husserl, 1982). By utilizing the epoché, we can shift our attention from *what* things are given in our experience, to *how* these things are given in experience, thereby adopting a *phenomenological attitude*.

From this transcendental standpoint, Husserl, similarly to Brentano, holds that acts of consciousness (i.e., perception, memory, imagination, etc.) are usually directed at something (perceptual objects, memories, expectations, etc.). This relation between acts of consciousness and objects of experience is named *intentionality*. This intentional relation between acts and objects is normative mainly because it implies what Husserl called a *structure of fulfillment* (Husserl, 2001, p. 280–283), which simply refers to how the intention of an act can be fulfilled by the intended object (Crowell, 2013; Doyon, 2015).

The intentional structure of fulfillment is particularly important for describing perceptual experiences, because the intention of perception is always directed toward a real and concrete object, and not merely to an imaginary or an abstract one. My visual perception of a tree intends the actual tree, not the concept or the re-presentation of a tree. However, perceptual objects will never fully fulfill my perceptual intentions because perceptual objects are always presented only partially (cf. Husserl, 2013). For instance, my perception of a tree from the window of my house presents only one sensorial profile of the tree (e.g., a couple of branches), whereas many other profiles remain hidden to my view (the backside of the branches, the trunk, the roots, etc.).

Despite this incomplete fulfillment, my perception is about the whole tree, not about one profile of the tree. This is nowadays called the problem of perceptual presence (Noë, 2004). This problem raises the question of (1) what conditions make possible that the sensorial givenness of only one profile of the tree evokes my experience of the tree as a unified whole; and also the question of (2) what makes one profile match with the anticipation of my intentional act that perceptually intends a tree. To respond to these questions, we need to clarify what the constitutive aspects of my perceptual experience are, as well as the character of the norm that relates the profile and the object.

The response of Merleau-Ponty to these questions originated in Husserl’s works^4^ is essentially that (1) the presence of perceptual objects as we perceive them is given thanks to a fundamental link between the bodily motor skills of a subject, and the motor significances of things. Merleau-Ponty called this link *motor intentionality* (Merleau-Ponty, 2012, p. 113). For Merleau-Ponty, the lived body of a subject implies an articulated unity that he called body schema (Merleau-Ponty, 2012, p. 100–103). This articulation is performed according to the needs of practical task. That is to say that the body schema is basically the self-organization of the body according to sensorimotor norms (cf. Gallagher, 2005). The lived thing, by contrast, is a unity of motor significations correlated with the motor skills of the body schema. We can interpret motor significations here as affordances. Hence the lived thing is the unity of affordances correlated with the motor skills of the body unified in the body schema (Merleau-Ponty, 2012, p. 334).

The synthesis of the thing (as a unity of affordances) is nonetheless a temporal synthesis or a synthesis of transition (Merleau-Ponty, 2012, p. 344). Only one profile of a thing is given at the present moment, however, the profiles non-viewed of the thing are lived as anticipations for motor actions. For instance, I cannot see the backside of my computer, but I can anticipate that if I turn it around, I will see its backside. This synthesis depends nonetheless on the synthesis of the body which is also temporal because the lived body is articulated thanks to its acquisition of bodily habits. These habits anticipate the encountering of perceived things in the way our body is familiarized to do it. The synthesis is, however, unfinished because both the body and the thing remain open to unexpected encounters, to failures in the norm that coordinate the movements of the body and the constraints of things (cf. Merleau-Ponty, 2012, p. 476).

Returning to the second of our earlier questions, (2) what allows for the disclosure of a thing, as a whole, from the sensorial givenness of only one of its profiles are the motor significations that such a profile affords to the body. Motor significations are invitations that the thing manifests or presents to my body from the current sensorial presence for exploring and manipulating it (Merleau-Ponty, 2012). The tree is given as a whole not because I imagine or represent the whole tree from my partial view of it, but because the tree itself affords further explorations to my motor skills, and its profiles, even those that remain invisible, are not really absent but *present* as correlates of my motor skills (Merleau-Ponty, 2012).

However, since things are never given as fully present, the possibility remains that my anticipations mismatch the actual conditions of things. Maybe if I get closer to the window, I can realize that the tree is not a real tree but a hologram of a tree, and then I won’t be able to touch it, to climb it, or to see its back, as I anticipate it. It was just an illusion. Perception, therefore, rests on anticipations that are never completely fulfilled. Nevertheless, there are still some angles or perspectives from where things are disclosed optimally. In this context, Merleau-Ponty claims that the optimum of perception (the optimal grip) is the way that an object present itself more clearly (Kelly, 2005), that is to say to find the right bodily articulation that better disclose the affordances of things (see section one). However, for Merleau-Ponty, the optimum (or the norm of perception) is not constituted by the characteristics of the thing itself, nor even by the relation between the body and the thing, but by the whole horizonal structure within which the body-thing correlation is enveloped (Merleau-Ponty, 2012).

A horizon is a phenomenological description of many structural aspects of experience that accompany the intended objects. For Husserl horizonal aspects or experiences are co-intended or co-given (Husserl, 1982, p. 94). Hence, horizons, roughly speaking, are those aspects of the perceptual field that play the role of a background for those objects I’m focusing my attention. However, horizons are more than a mere accompaniment to focused objects; they are a constitutive part of my lived experience of them. For this reason, Merleau-Ponty holds that the optimum of perception involves the equilibrium of internal and external horizons (Merleau-Ponty, 2012, 316). Husserl defines the internal horizons of perceptual objects as those aspects that are not directly intended, but are still part of the intended object, as with the unseen profiles of the tree. The external horizons, by contrast, are those elements that surrounds the object, like the garden where the tree is rooted, the blue sky that contrasts with the green of its leaves, etc. (Husserl, 1982).

Horizonal aspects of experience are not only those elements implicit in the perceptual field, but also the motor skills that correlate the motor significations of things. That is why Merleau-Ponty claims that the body is the third element implicit in the figure-ground couple of perception (Merleau-Ponty, 2012, p. 103). However, these bodily skills are constrained by the whole relation of forces present in the field. As Merleau-Ponty claimed in *The Structure of Behavior* (Merleau-Ponty, 1963), the soccer field, for the player, is not an object but a field of forces where consciousness consists in the dialectics between the milieu and the body. Moreover, this field constantly changes considering the actions accomplished by the body, establishing new lines of forces (Merleau-Ponty, 1963, p. 168–169). Therefore, the norms of perception are not constituted by the characteristics of things as such, so much as these characteristics are implicitly correlated to the abilities and skills of the body, and all those horizonal aspects that structure the whole condition of the phenomenal field where any focused aspect is always embedded.

The whole normative framework of perceptual experiences is thus ecological because such a framework depends on structures present in the environment that constitute the way an object can be optimally disclosed by a perceiver. The adjective ecological, in this case, does not only implicate the relation between an agent and the environment, as ecological approaches affirm, but also the subjective engagement of an agent into its environment. That is, how the environment appears for the agent according to its embodied subjectivity.

The optimum is thus the norm of a whole situation that can involve many worldly aspects that constitute the forces of the field, but that can be also altered, changing the orientation of these forces, manifested in a new sense of perceptual experiences. To improve our understanding of these perceptual norms, Merleau-Ponty’s scholars have been lately appealing to the notion of spatial levels that I will review in the next section.

### Levels of Perception

In his *Phenomenology of Perception*, Merleau-Ponty describes the general notion of space from a phenomenological standpoint, including the body as a constitutional aspect of this dimension. Merleau-Ponty highlights that our experience of space, in normal conditions, implies a particular orientation (e.g., up, down, left, right) that is given to us without the need of conscious reflection (Merleau-Ponty, 2012, p. 259). The primordial sense of space, for Merleau-Ponty, is not an abstract geometrical dimension that works as a sort of container for the objects and events that exist in the world, which is what Merleau-Ponty calls *positional spatiality* (Merleau-Ponty, 2012, p. 102). Rather, for Merleau-Ponty, the primordial form of spatiality is a *situational spatiality* that involves the active engagement of the body in the accomplishment of motor tasks (Merleau-Ponty, 2012, p. 102). This general notion of space entails the horizonal domain of all our possible bodily actions (Merleau-Ponty, 2012, p. 260).

There are more concrete or delimited spatial regions that following Casey (1996, 1998) we can call *places*. These places possess *anchorage points* that allow our bodies to situate themselves in or inhabit them (Casey, 1998, 229; Merleau-Ponty, 2012, p. 259). The anchorage points grant places a kind of stability, establishing what Merleau-Ponty called *spatial levels* (Merleau-Ponty, 2012, p. 259). These levels are normative aspects of perception because they refer to the habitual or preferential ways our body interacts with the environment, something that presupposes a previous attunement or “a pact,” between the body and world (Merleau-Ponty, 2012, p. 261).

The kind of normative character of levels thus does not refer to the perception of things, but to the horizonal aspects that accompany it. Talero, for instance, claims that a spatial level “establishes a place or a setting for my actions to range over by inaugurating a preferential perceptual norm within my situational spatiality” (Talero, 2005, p. 448). That is, *levels are norms of Places*. Unlike things, places are not usually the focus of our attention. Instead, places tend to serve as stable settings that background our everyday activities and aspects of the environment that we find relevant (e.g., things, colors, shapes, etc.). Hence, places, from a phenomenological standpoint, are horizonal aspects of our perceptual intentions that work as the counterpart of our embodied subjectivity.

The ubiquitous presence of some spatial levels (the more general ones) requires that we alter the normal conditions of our sensorimotor interactions to be able to recognize them. This is what Merleau-Ponty did through his interpretation of a few classical experiments. First, he refers to a Stratton’s experiment where a subject use goggles that invert the visual field for 8 days. The visual field is perceived up-side down at the beginning. After a couple of days of use, however, the subject starts to live the visual field normally but begins to feel that her body is inverted. After 8 days of use, the whole sensorimotor interaction is finally readapted, and the visual field is lived normally (Merleau-Ponty, 2012, p. 255). In the second example, Merleau-Ponty describes Wertheimer’s experiment, where a subject is put in a room, but can only see through a mirror that is tilted at an angle of 45 degrees. The subject initially sees everything obliquely, and even the movement of objects in the visual field is perceived with an oblique deviation. However, after a few minutes, the subject starts to perceive the entire scene vertically once again (Merleau-Ponty, 2012, p. 259).

These examples allowed Merleau-Ponty to see that spatial levels exist in our normal sensorimotor coupling, and that some habitual sensorimotor coupling can be altered if we modify the feedback of “normal” sensorimotor loops. Levels thereby are not properties of the environment as such, nor a projection of agents, rather they describe the normative entanglement of both, but more importantly they also describe the open-ended character of the normative frameworks of action and perception that constantly evolve. This is a phenomenon that (*Level shifts* called Talero, 2005, p. 446).

In level shifts, it is common that the anchorage points or the structure of the original level is transposed into a new level, just as when we transpose a melody from one tonality to another (cf. Merleau-Ponty, 1963, p. 87). This kind of transposition of levels explain why our bodies can become geared, in the same manner, to different environments with similar structures. In the two experiments referred by Merleau-Ponty, the sensorimotor loop is altered but the same form is perceived. From a phenomenological standpoint, this happens because our perceptions are based on anticipations of potentialities for motor actions, but critically these anticipations are grounded on the sensorimotor habits previously acquired by the perceiver. These habits are correlated to the motor significations perceived in the environment and are anticipations and motivations for motor action. Therefore, when a level is forced to shift into another level by changes induced in the sensorimotor loop, the body aims to use its habitual sensorimotor coordination, but is forced to reorganize this coordination considering the new circumstances. However, since it is possible to find similar anchorage points in the emergent sensorimotor dynamics, the habitual form can be transposed into the new level.

The crucial aspect of this description of levels is their open-ended character, which is not only exhibited by the experiments from above, but seems to be a necessary condition for explaining why the interactions between the body and the environment always remain open to continuous readjustments that nonetheless follow predictable paths inherent to the normative frameworks of levels previously enacted (cf. Merleau-Ponty, 2013, p. 76). The description of levels is ultimately a description of an endogenous developmental process of the agent-environment entanglement. Considering this description of levels, as the normative framework of space and places, our last task is to clarify how levels contribute to our understanding of norm-development.

### The Development of Enactive-Ecological Norms

The ecological approach of the skilled intentionality framework analyzes the norms of action and perception in terms of what I called normative frameworks (situated normativity) and norm-attunement (skilled intentionality). From this viewpoint, individual agents become attuned to pre-established normative frameworks. The problem with this viewpoint lies in the way it describes normative frameworks as constituted independently of individual agents, and before these agents are engaged in bodily practices.

The enactive approach, by contrast, is capable of a more adequate account of the continuous development of the norms of practices. Norm-enactment, contrary to norm-attunement, involves the active participation of agents in the constitution and development of norms. However, the enactive approach sometimes reduces its account of norms to a relation between autonomous agents and physical constraints, thereby neglecting the existence of normative frameworks that constrain the enactment of norms.

I argued above that norm-enactment is not simply the projection of meaning to physical reality, nor can such enactment be understood as the emergence of meaning from mere physical constraints. Rather, norm-enactment entails the constant development of norms from previously given normative frameworks, something I called norm-development. The account of sensorimotor norms of the enactive approach points to the description of this phenomenon. Nonetheless, this account is still insufficient, because it ignores the fact that norm-development does not simply involve the development of bodily habits, but also the development of environmental structures that embody the counterparts of bodily habits.

To improve our understanding of this dynamic of norm-development, I appealed to the phenomenological account of perceptual norms found in the writings of Merleau-Ponty, and to his concept of spatial levels. From a phenomenological perspective, norms of perception are moments of equilibrium between the whole ecological context (a situation) and the embodied subjectivity of an agent, instituting what Merleau-Ponty called levels. These levels, however, are in constant development (level shift) because the agent-environment entanglement is a temporal and open-ended structure that is in a constant conflict and movement.

Norm-development, however, is not purely dynamic phenomenon. It also implicates the stability of norms as horizonal normative frameworks. On the side of the agent, this stability is incarnated in bodily habits, while on the side of the environment, stability is expressed as what I described as excorporations. These are anchor points of places that enable and constrain the enactment of more specific aspects of the environment, which we can understand as affordances. Excorporations may relate to the concept of ecological information in the ecological tradition, whereas levels point to the normative frameworks that constrain the enactment of new norms of action and perception.

## Conclusion

The norms of action and perception are not pregiven sets of lawful relations, nor static frameworks that constrain the behavior of agents until we consciously change them to become adapted to the new worldly circumstances. All bodily practices are highly dynamic, our bodies, the environment, our relations with others, are constantly flowing processes that nonetheless find periodical moments of stability. Stability and change are the two crucial features of life and cognition, either from a dynamical systems theory perspective or from a phenomenological analysis. If we failed to acknowledge one of these aspects, we will fail to describe accurately the dynamics of life and cognition. We must therefore construct an approach to norms of action and perception that acknowledge these two central features of norms.

I proposed an enactive-ecological approach to norms. This approach is based on the process of norm-enactment described by the enactive approach, but that incorporates an account of normative frameworks. Since these frameworks are not accurately described by the skilled intentionality framework, I appealed to Merleau-Ponty’s phenomenology for this task. The notion of spatial levels broadly describes temporally open-ended normative frameworks that constrain bodily practices but also make possible the enactment of new norms for these practices. I named norm-development as the process of norm-enactment that implicates the temporal evolution of normative frameworks. A more detailed descriptions of norm-development is still needed, as well as the way to apply these descriptions to the concrete normative domains of interaction of agent(s)-environment systems.

These conclusions, in favor of an enactive model over an ecological one, must not let us think that ecological approaches, specially the skilled intentionality framework, are not highly valuable for our study of cognition from a radical embodied cognition perspective. Rather, if my arguments are right, this is a call for ecological approaches to become more truly enactive. Many of the concepts of the skilled intentionality framework and the free energy principle that support this theory are already pointing in this direction (Kiverstein and Rietveld, 2018).

Although at a high theoretical level, it is still hard to see a real complementarity between ecological and enactive approaches, their few but crucial discrepancies are currently useful to create a productive dialogue between these two radical forms of embodied cognition. I hope the reader has found in this work a nice example of it.

## Author Contributions

The author confirms being the sole contributor of this work and has approved it for publication.

## Conflict of Interest

The authors declare that the research was conducted in the absence of any commercial or financial relationships that could be construed as a potential conflict of interest.

## Footnotes

1 In the more specific context of 4e cognition, some philosophers influenced by Wittgenstein’s late philosophy insist that norms and normativity apply only to actions concerning sociocultural practices, because only these practices implicate a criterion of correction that is agreed by a community (Heras-Escribano et al., 2015). The supporters of the enactive approach hold a wider conception of norms that include multiple aspects of life and sensorimotor interactions between biological agents and the environment (Barandiaran and Egbert, 2014). I depart from this general definition because later, the more specific conceptions of norms of the skilled intentionality framework, the enactive approach, and Merleau-Ponty’s phenomenology will be defined.

2 In physics, the second law of thermodynamics states that all physical systems have the tendency to increase chaos and disorder, which is analogous to saying that systems have the tendency to reduce (thermodynamical) free energy. Claude Shannon made a similar claim for his theory of information, positing that all informational systems have the tendency to reduce uncertainty in the same lawful manner that physical systems reduce thermodynamical free energy. In the case of informational systems like cognitive systems, however, we are talking about variational, rather than thermodynamical free energy (Kirchhoff and Froese, 2017).

3 However, the specific notion of active inference, part of the conceptual repertoire of the skilled intentionality framework, may suggest that such development occurs (cf. Ramstead et al., 2019).

4 The normative condition of experience and more particularly of perception was originated in Husserl’s work (Crowell, 2013; Doyon, 2015, 2019). It was Merleau-Ponty, however, who more systematically develop this subject. Since I find relevant the notion of spatial levels from Merleau-Ponty’s phenomenology to improve our understanding of norm-development, I will refer almost exclusively to Merleau-Ponty’s work.
